# How Do Men Become Doctors?

**Published:** 1884-01

**Authors:** Ben. H. Pratt


### THE BISTOURY.

Vol. XIX. ELMIRA, N. Y., JANUARY, 1884. No. 4.

### Contributions.

### HOW DO MEN BECOME DOCTORS?

BEN H. PRATT.

I have many times set myself the task of solving the question, "How do men ever reach the determination to become doctors?"

I have never been able to answer the question satisfactorily to my own mind. There may be a lack of the reasoning faculty necessary to reach that conclusion from data at hand. It is possible that I have never gathered all the data that should enter into the consideration of the subject. Again, I may have made wrong estimates in my summing up of the faculties of the human mind that would naturally be called into action in arriving at such a determination; or I may have over-estimated some of these, or under-estimated some.

I find, in my inventory of the average human mind, one or two very stubborn barriers to the decision in question. I find, among others, conscience, caution and policy. Every man that I know anything about has a conscience. He may crave money, reputation and recognized position, but under ordinary circumstances he doesn't desire these at the expense of not deserving them. When he deliberates upon assuming the role of a healer of the ills to which modern flesh is heir, he is tackling a mighty matter. It seems to me that the young man who revolves this question in his mind has about all he wants to do for the time being. He must reflect that success in the healing art implies great wisdom, wide learning, peculiar tact, vast patience, long application, rare judgment, uncommon discretion, good guessing faculties, plausible inventive genius or powers of invention, vivid imagination, quickness of perception, coolness under the most exciting situations, angelic amiability, sublime expediency, moral and physical courage, skill in ambiguous expression, deference to the crankiest of opinions, whims and notions, cheerfulness amid the gloomiest surroundings—in fact, one finally reaches the conclusion that it requires something a little beyond the ordinary mortal of our species to make a doctor that would satisfy the average conscience. Now there are very few men who have the audacity or the ignorance or the baseness to admit that they possess enough of the above named qualities, and in such a degree as will admit of their entering upon the practice of medicine. But we have a great many doctors, nevertheless, and it is the attempt at the reconciling of the latter fact with the requirements named, that makes me wonder how all these men ever overcame all these barriers and are all apparently successful practitioners to-day.

I don't like to think that these men have either very little conscience; or that they haven't allowed their conscience to have much to do with the matter; or that they have the assurance to think that they possess the qualifications named. But I can't reconcile the results with any theories I have, and long ago gave it up.

It seems to me that some such procession of thought as this must pass through the mind of the young man who is contemplating the choice of medicine as his life work.

If I enter upon this profession I must not only master the technical details thereof, but I must accept terrible responsibilities. The lives of precious human beings will be confided to my keeping. To the ordinary ills of the human family I may perhaps bring all the needed remedies. But some day I am sure to be called to the bedside of some one whose malady I may be unable to know; whose tongue and pulse and symptoms conflict with all the recorded cases on my shelves, and with all those that have ever come within my experience. How am I to make a diagnosis in such a case as this? And of what earthly use is a doctor without a diagnosis? Having none, what am I to do with this patient? Perhaps, had I not undertaken this profession, some more learned, more instinctively acute, more skillful disease-reader would have been called to this case, and would have known what to do for it. I do not know and therefore I must make a guess. On this guess may depend the life of this patient. If it's a good guess, it's my luck; but what if it is a bad guess? Will I be less a murderer, if that patient dies which some other man might have healed, than if I had stabbed him to death? Isn't it as bad to let die as to kill? Having made my guess, and it being only a guess, what I do in the after proceeding upon that guess is, of course, peradventure. If my patient recovers it will only prove that I guessed pretty well. If he die, a *post-mortem* may reveal my ignorance, my lack of judgment, my tendency to guess and to act upon a guess, and hence reveal the fact that I had no business to study medicine, a conclusion that should have come to my rescue before I entered upon the practice of that art, or science.

It is scarcely admissible that a young man will plunge into the study of medicine, or of anything else, without some thought—without forecasting possibilities and probabilities—without dwelling at considerable length, and with much seriousness upon the work before him. In this forecast of his career it is reasonable to suppose that he will not regard himself as superhumanly endowed, but will admit that he is liable to err in judgment, and that he is not capable of achieving a more thorough knowledge of the art of healing than hundreds of doctors with whose careers he is more or less familiar. He will naturally picture himself a full-fledged physician on his daily rounds in response to the calls of his "large and lucrative practice." Among possibilities—not only that, but among probabilities—I might say, among absolute certainties that he will be called upon to confront, will be something like this, which may be put in the form of

THE MEDICAL STUDENT'S SOLILOQUY.

I will be called to see Smith, summoned by a telephone message delivered in such a tone and manner as to lead me to understand that Smith is in a very bad way. I am Smith's family physician. He is a profitable patient. I have long since secured the complete confidence of the Smith family. They think I am the only doctor in the world who thoroughly understands the idiosyncrasies of every member of the family, from Smith, Sr., the present prostrate, down through madam and the masters and the misses Smith, to and including the last puling Smith in long clothes.

I am met at the door by the anxious, yet almost hopefully radiant faces of the numerous Smiths, masculine and feminine, and I realize that at last I have a serious case on my hands. I am ushered into Smith's chamber, followed by the family procession, which gives me five minutes to decide "what is the matter with husband?" "What ails pa?" I find Smith's tongue indicates one thing, his pulse another, his temperature another, his respiration another and his symptoms a whole host of other distinct troubles, all of which he can't possibly have, any one of which he may have, complicated with a larger or smaller number of the almost infinite remainder. Lacking omniscience, and possessing clairvoyant powers in a very moderate degree, I hesitate to say, at once what the matter with pa is. Heretofore I have had plain cases in the family, and have been in the habit of telling them at once what the trouble was with any of them. I have thus acquired a sort of oracular position in the family. Under the present circumstances I can't give an opinion at once. In other words, I am stuck. I have never seen anything just like, nor anywhere near like this case. The family confidence in me weakens at once. I see them look at each other and at Smith and at me in a way that doctors don't enjoy having their patient's people look. I want time for this case, and because I don't hazard an opinion at once, a dreadful suspense seizes the whole family, and this becoming intensified as the moments of my indecision pass, I am likely to have some cases of hysteria or syncope on my hands within a very few minutes, for the best of this Smith family isn't robust, and some of it is shockingly delicate. I continue the examination of my patient—as kindly as I can ask that the family retire for a few moments, ostensibly, that I may make a more critical examinations but really to be relieved of the embarrassing questions as to pa's trouble. But I've made a great mistake. My sending out of the family has aroused fears that something terrible is the matter with pa; that he is so very bad that I don't dare tell how bad, and he is believed about to depart life very shortly. They can't conceive now that I don't know what's the matter. It never enters the heads of the household that their family physician may not know all about his patient the minute he has seen the tongue and felt the pulse and asked three questions, and coughed and said humm. I, their doctor, in their belief, won't tell because what I have to tell is too bad to be told, and the family gathers in the next room, and I can hear them sobbing and oh-dearing, and what's-to-become-of-us-ing, and poor-dear-pa-ing, and why-didn't-we-call-the-doctor-sooner-ing, all of which does not have a tendency to put my mind into any sort of condition for calmly meditating upon the conflicting symptoms before referred to. I mechanically go on with my examination of the patient, however, and the more I learn the less I know, for in all my books and in all my practice (imaginary) I have never come across such a case as this. And when the family comes back into the sick chamber with their red eyes, and blowing their noses and heaving great locomotive sighs, it is very evident that they have become reconciled to the situation and have it all figured out in just what order they will walk in the funeral procession. I am still necessarily silent on the cause of the difficulty. I am afraid to give an opinion in the case, for I don't know how soon a consultation may be asked for, and some other doctor politely call me a liar by pronouncing the disease something entirely different. But I finally get away from the patient, leaving him with a goodly array of drops and powders, and a long series of directions how to proceed during my absence, and I rush toward home to find something in my books that will give me some clue to the case in hand. The family follows me to the door with tear-filled eyes desiring to know how soon the terrible blow is liable to fall upon the household, and I go out in a chorus of sobs. On my way home I wonder how I could have been so lackwitted as not to give the malady a name—that name which has come as a God-send to the profession, embracing everything not embracable under any of the other recognized forms of disease. I smite myself metaphorically, for not having thought to confidently declare the case one of those malarial attacks which of late exhibit themselves under such varied forms that it is impossible to determine the precise seat of the disease, but which a few hours, under a special form of treatment, will develop clearly. I might have saved all this lachrymose business. I might have saved myself a vast amount of embarrassment, and I would have been perfectly safe in asserting the malarial origin of the trouble, for that doctor does not live who could ever have proved, ante-mortem or post-mortem, that what ever Smith may have had, it wasn't a malarial something or other. But I determine to fix it when I return. When I go back to my patient, it is only to find that Smith was in the rigors of an old fashioned fever and ague attack, and next day he was down at his office as well as ever.

But I, the doctor, what of me? How narrow an escape I have had. And isn't it now being canvassed all about the community that I couldn't tell a case of old fashioned ague from a malarial something or other? And isn't it rather significant that I am not the oracle, especially in the Smith family, that I once was? All this, to say nothing of the wear and tear of conscience that I have gone through in this one case.

What is there to prevent the young man who thinks, and has a conscience, and is endowed with a reasonable caution, from dwelling upon his prospective calling and conjuring up just such possibilities as this? The contingencies are fearful to contemplate, and he is a bold youth, and a self-confident youth, and a reckless youth that will not, after such contemplation, wheel off into some less hazardous calling, one with fewer awful responsibilities attached.

"How do men ever become doctors!" is a good deal like the question, "how do boys ever grow up to be men?" With all the contingencies that lie in the pathway from the boy's babyhood to his manhood, it is a marvelous fact that thirty-three and a third per cent. of the boy babies born into the world get to be twenty-one years old, despite the traps and snares and dangers of every sort that the average boy seems to be constantly surrounded by until he reaches his majority. But he reaches it, somehow; and young men determine to be doctors, somehow; and they get along and are apparently as happy and as much thought of as other people.

I often look at young medical students, and at young doctors with new signs nailed up on their office doors, and wonder how they ever got through with the mental struggle that finally resulted in their becoming doctors. Some of them show the evidences of the conflict in their yet agonized features, while some look as though they had never a misgiving on this or any other point. Doubtless many plunged into the profession without any serious contemplation of results, perfectly willing to abide by what comes when it comes. I can't see how, otherwise, they ever reached their degrees.

I find, too, scattered all through the communities in which I have mingled, a greater or less number of men who are called doctors, but who are not practicing medicine, and so far as I can tell, never have practiced the art. They are in other kinds of business, but their titles hang on very much as the titles of colonel and major and captain and judge that many men have, and the origin of which we never think of tracing up. These doctors are evidently men who plunged into the profession without consideration of the coming responsibilities, and after they became doctors first realized the fearful situation in which they had placed themselves, declined to accept the hazards, and gracefully withdrew into other and less responsible paths of life.

It is, perhaps, well that there are men, young men, with sufficient self-confidence to enable them to determine to be doctors. It is, possibly, a fortunate thing that all do not stop at the threshold of medicine to count the contingencies within that prefession. If all did so, doubtless there would be a fearful scarcity of doctors, and every doctor would be a monopolist. It is even more disagreeable to contemplate such a condition of things than to realize the present apparent superfluity in the profession.

It is likely that it is all for the best that there should be as many as there are who do not hesitate to assume all the responsibilities and take all the risks of becoming doctors, but *how* they manage to do it remains, all the same, one of the preplexing questions of the age.

